# Application of bioreactor technology for cell culture-based viral vaccine production: Present status and future prospects

**DOI:** 10.3389/fbioe.2022.921755

**Published:** 2022-08-09

**Authors:** Zhongbiao Fang, Jingting Lyu, Jianhua Li, Chaonan Li, Yuxuan Zhang, Yikai Guo, Ying Wang, Yanjun Zhang, Keda Chen

**Affiliations:** ^1^ Shulan International Medical College, Zhejiang Shuren University, Hangzhou, China; ^2^ Zhejiang Provincial Center for Disease Control and Prevention, Hangzhou, China

**Keywords:** bioreactor, viral vaccine production, cell culture, process optimization, computational biology, COVID-19

## Abstract

Bioreactors are widely used in cell culture-based viral vaccine production, especially during the coronavirus disease 2019 (COVID-19) pandemic. In this context, the development and application of bioreactors can provide more efficient and cost-effective vaccine production to meet the global vaccine demand. The production of viral vaccines is inseparable from the development of upstream biological processes. In particular, exploration at the laboratory-scale is urgently required for further development. Therefore, it is necessary to evaluate the existing upstream biological processes, to enable the selection of pilot-scale conditions for academic and industrial scientists to maximize the yield and quality of vaccine development and production. Reviewing methods for optimizing the upstream process of virus vaccine production, this review discusses the bioreactor concepts, significant parameters and operational strategies related to large-scale amplification of virus. On this basis, a comprehensive analysis and evaluation of the various process optimization methods for the production of various viruses (SARS-CoV-2, Influenza virus, Tropical virus, Enterovirus, Rabies virus) in bioreactors is presented. Meanwhile, the types of viral vaccines are briefly introduced, and the established animal cell lines for vaccine production are described. In addition, it is emphasized that the co-development of bioreactor and computational biology is urgently needed to meet the challenges posed by the differences in upstream production scales between the laboratory and industry.

## 1 Introduction

**FIGURE 1:**
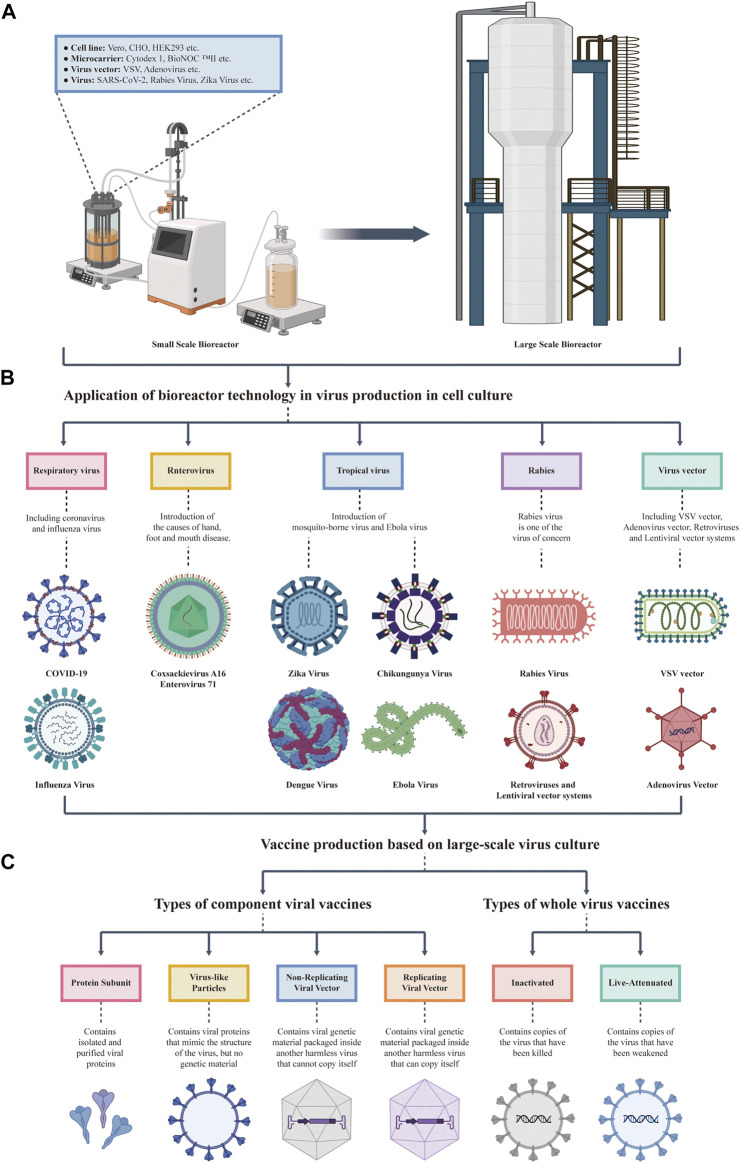
Overview the upstream culture process of viral vaccines. **(A)** Development and scale-up of a cell culture bioreactor. The left plot shows a small-scale bioreactor, which can contain cell lines, microcarriers, virus vectors, and viruses. The right plot shows an engineering grade bioreactor that can be produced on a large scale at one time. **(B)** Virus produced by cell lines in a bioreactor. The types of viruses produced by cell culture in a bioreactor mentioned in this review, including SARS-CoV-2, Influenza Virus, Tropical Virus, Enterovirus, and Rabies Virus. **(C)** Vaccine production based on large-scale virus culture. Bioreactors are mainly used to produce inactivated vaccines, live attenuated vaccines, and several subtypes of vaccines (including protein subunit vaccines, virus-like particles vaccines, and replicating viral vector vaccines).

Bioreactors play an important role in the production of large-scale viral vaccines in cell culture (Figure 1). With the advance of vaccine production process, increasing numbers of scalable bioreactors and cell lines with high affinity for viruses have been applied for the production of different vaccines. In 1962, Capstick et al. domesticated BHK21 cells to achieve suspension culture and applied them to veterinary vaccine production (Capstick et al., 1962). In 1967, VanWezel developed microcarriers and achieved the culture of adherent cells in a bioreactor (van Wezel, 1967). The development of suspension and carrier cultures in bioreactors marked the beginning of large-scale cell culture. After the 1980s, CHO cells were cultured in suspension. The development of therapeutic antibody production technology has markedly promoted the application of bioreactors in the biopharmaceutical industry. By the end of the 20th century, it reached a scale of 10,000 L (Griffiths, 1990). Currently, with the development of fed-batch culture (Gutiérrez-Granados et al., 2018), perfusion culture (Teworte et al., 2021), and genetic engineering (Pino et al., 2020), bioreactors have developed into a platform in which cells are used to produce a variety of viral vectors, live viruses, and virus-based vaccines. These bioreactors combine the low shear environment of traditional systems with the scalability of automated systems, and have a broader application prospect (Von den Eichen et al., 2021).

To date, emerging infectious diseases have seriously affected social stability and have posed a significant threat to human health. In particular, coronavirus disease 2019 (COVID-19) caused by Severe Acute Respiratory Syndrome Coronavirus 2 (SARS-CoV-2), has spread worldwide (World Health Organization, 2020). In the face of the COVID-19 pandemic, vaccine development has been ramped up to an unprecedented speed. By 4 April 2022, 11.25 billion doses of vaccine had been administered worldwide (World Health Organization, 2022a). Public health experts estimate that only a very high level of SARS-CoV-2 vaccination can induce mass immunity (Carpio et al., 2021a; Carpio et al., 2021b). However, as the virus rages, the economies of many low-income countries have been seriously affected. This leads to limited funding for vaccine production and procurement, causing an uneven distribution of vaccines worldwide (Sawal et al., 2021). In addition, in the production of mRNA vaccines, the lack of basic raw materials and the difficulty in increasing the scale of production are also the main bottlenecks in the production of such vaccines against the novel coronavirus at present (Kis et al., 2020). Compared with mRNA vaccines, vaccines produced by traditional whole virus inactivation technology are easier to distribute and store because they only need to be refrigerated; however, the production of inactivated vaccines cannot meet the requirements of group immunity (Risson, 2020).

For most pathogens similar to the novel coronavirus, large-scale vaccine production is very important for global disease control and eradication, because the high mutation rate of the virus might lead to a decrease in the protection offered by the vaccines (Bakhshandeh et al., 2021). Therefore, research on the production and culture of virus vaccines, especially exploration at the laboratory scale is urgent needed. Using bioreactors to culture cells to produce antigens, antibodies, and other products is the core technology underpinning the large-scale production of biological products. The combination of fine process control in biotechnology and the screening and domestication of high-expression cell lines can improve production efficiency and product quality, and reduce cost (Pau et al., 2001; Pan et al., 2019; Shen et al., 2019). Based laboratory scale experiments, we can optimize some conventional physical and chemical parameters and obtain a more perfect training program on a larger scale in advance, which saves time during subsequent industrialization. More importantly, laboratory scale experiments can provide pilot-scale conditions for new biological products, especially in the research and development of new vaccines, to maximize the yield and quality.

This review summarizes the application of bioreactor technology in the production of cell culture virus vaccines. First, we introduce the different types of viral vaccines produced at present, and describe the animal cell lines that have been established for vaccine production. Then, the main types of bioreactors are introduced, and the important parameters related to large-scale virus amplification and their effects on virus yield are summarized. On this basis, we systematically evaluated the process optimization methods for the production of different viruses (SARS-CoV-2, Influenza Virus, Tropical Virus, Enterovirus, and Rabies Virus) using bioreactors (Figure 1B, Table 2), providing valuable information for the laboratory simulation of vaccine production and industrial large-scale vaccine production. At the same time, many studies have found that SARS-CoV-2 has similar characteristics to influenza virus and rabies virus in different cell models (Gao et al., 2020a; Wang et al., 2020a). Therefore, this review might also provide a variety of effective ways to develop cell culture process for the large-scale production of SARS-CoV-2 vaccines. In addition, the topic of mutual development of bioreactor and computational biology are also discussed to meet the challenges posed by differences in the scale of upstream production from laboratory to industry.

## 2 Bioreactor-based vaccine manufacturing

### 2.1 Types of vaccine

Among the many kinds of vaccines, the mainstream vaccines for viral pathogens can be divided into two general types: inactivated vaccines and live attenuated vaccines. Within this generally accepted classification, different technological approaches can be applied, giving rise to several sub-type vaccines, such as protein subunit vaccines, virus-like particles vaccines, replicating viral vector vaccines, and nuclear vaccines (Figure 1C). Different technological approaches of these vaccines are described as follows:

• Protein subunit vaccines elicit an immune response by employing viral proteins or protein fragments, based on synthetic peptides or recombinant proteins (An et al., 2022).
• Virus-like particles vaccines contain viral proteins that mimic the structure of the virus, but no genetic material (Haynes, 2009).
• Viral vector vaccines use a non-replicating virus to deliver DNA containing viral genes to human cells (van Doremalen et al., 2020).
• Inactivated vaccines incubated the virus in continuous cell lines or tissues before purifying, concentrating, and inactivating the virus (Wang et al., 2020b).
• Live-attenuated vaccines eliminate specific viral components or use codon optimization to reduce toxicity while preserving immunogenicity (Lauring et al., 2010).

When a virus spreads and mutates, new strains can emerge that are not protected by the vaccines that are already available, which can lead to changes in the protection effectiveness of different types of vaccines (Li et al., 2022). Viruses with a high mutation rate are unsuitable for live attenuated vaccines, as their mutations can be reversed during vaccine production (Lauring et al., 2010). In addition, the replication of attenuated virus strains in cell culture may be diminished, resulting in a decrease in process output (Zamarin et al., 2006). In contrast, Inactivated vaccines made with wildtype viruses yield more virus, but High-pathogenic wildtype live viruses may require biosafety level 3 conditions (Abdoli et al., 2021). The screening and domestication of high expression cell lines as an important part of large-scale vaccine production in cell culture, which will be introduced in the next section.

### 2.2 Selection and development of cell lines

The development of cell culture systems for virus transmission has promoted the development of viral vaccines. Chicken embryo fibroblasts in primary culture are often used in the manufacture of human vaccines (Gallo-Ramírez et al., 2015). At present, continuous cell lines overcome the shortcomings of primary cell lines and can adapt to modern cell culture techniques, which mainly use “designed cell lines” to increase virus production. These designe cell have been carefully defined and were generated particularly as a cell substrate for one purpose (Genzel, 2015).

The Vero cell line can proliferate only when there is a suitable surface of a microcarrier (Emeny and Morgan, 1979). Preflucel ^®^ is an inactivated seasonal influenza vaccine produced by Baxter, based on Vero cells, and was licensed by the European Union in 2010 (Chan and Tambyah, 2012). The vaccine was made from three strains of H1N1, H3N2, and influenza B vaccine. Vero cells were grown on Cytodex three microcarriers. A recent study using vesicular stomatitis virus (VSV) as a model found that the maximum cell density of Vero cells suspended in serum-free medium (SFM) was similar to or better than that of Vero cells observed in commercial SFM. That is, higher cell density (8 × 10^6^ cells/mL) could be obtained in IHMO3 medium (Shen et al., 2019).

Madin-Darby canine kidney (MDCK) has become the main suspension cell line in influenza vaccine production (Bissinger et al., 2019). At present, MDCK suspension cells are expanded in a bioreactor with an alternating tangential flow (ATF) perfusion system. And the highest virus titer of influenza A virus at 4.37 log10 (hemagglutination units (HAU)/100 μL) and infectious virus titer 1.83 × 10^10^ virions/mL were maintained (Wu et al., 2021).

PER.C6 cells can also be suspended to a high density (up to 10^7^ cells/mL) in SFM for a short time, without any solid support, and they are sensitive to all influenza virus strains. Therefore, the PER.C6 cell line is also being used in influenza vaccine production (Pau et al., 2001).

The Chinese hamster ovary (CHO) cell line is a popular new “designed cell line” (Geisse and Voedisch, 2012), and the improvement of CHO cell culture technology is also the mainstream direction of current research. For instance, Schmitz J et al. (Schmitz et al., 2021) demonstrated that MSCC facilitates small-scale culture of mammalian cells in terms of specific growth rates, cell diameters, and eGFP yields. And this has important implications in the cell culture stage of vaccine production. In addition, in the CHOBC clone expressing hIgG1, the ActiCHO process was used and compared with the traditional process, the cell volume and the titer of monoclonal antibody increased significantly in the ActiCHO process (Pan et al., 2019). The CHO cell line has a complex glycosylation system, which is helpful for the stable expression of the SARS-CoV-2. The CHO cell line has a complex glycosylation system, which is helpful for the stable expression of the SARS-COV-2, which can increase the content of the glycosylated spike (S) protein with higher sensitivity and specificity for antibody detection (Pino et al., 2020). Recently, the COVID-19 recombinant protein vaccine jointly developed by the Institute of Microbiology, Chinese Academy of Sciences and Anhui Zhifei Longkoma Biopharmaceutical Co., Ltd. is a CHO cell-produced vaccine (Liu et al., 2021). Esposito et al. (2020) showed that the yield of Vaccine Research Center (VRC) S protein was higher than 5 mg/L when captured using an immobilized metal ion affinity chromatography (IMAC) column and purified by desalination column at 32°C for 96 h.

The human embryonic kidney cell line, HEK293, is the main cell line for transient expression of recombinant proteins (Geisse and Voedisch, 2012). HEK293 cells were immortalized by transfecting cleaved human adenovirus 5 (Ad5). The genes of adenovirus proteins E1A and E1B are integrated in its genome (Douglas and Quinlan, 1995). The expression of these two proteins can promote the growth of HEK293 cells by regulating cell cycle and apoptosis (Spitkovsky et al., 1994). Expi293FTM is also used to produce novel coronavirus vaccines. At present, using conventional transfection reagents and schemes, the final yield of receptor-binding domain (RBD) is 90 mg/L (Castro et al., 2021).

Each virus has its optimal celline for growth, and human diploid cells such as medical research council cell strain-5 (MRC-5) are the most suitable celline because of genetic stability and the less adverse reactions for human use. Although human diploid cell culture is suitable for vaccine, it is not ideal for large-scale culture and the cell density cannot break through a bottleneck.

## 3 Development and process optimization of bioreactors for virus production

### 3.1 Types of bioreactors

Traditionally, Embryonic stem cells (Ying et al., 2003), induced pluripotent stem cells (D'Aiuto et al., 2014), mesenchymal stem cells (Moreira et al., 2020), and other stem cells were all expanded and subcultured in the presence of serum and a feeder layer, comprising the standard two-dimensional (2D) culture model (Freshney, 2015). This method needs to separate the feeder layer; however, there is a risk of pathogen contamination and it is not easy to operate (Freshney, 2015). The cultured stem cells are prone to variation and the cell yield is also limited (Ying et al., 2003; D'Aiuto et al., 2014; Moreira et al., 2020). The use of a bioreactor solves these problems to some extent. The design concept of early bioreactors was mainly based on stirring bioreactors for microbial fermentation (Garcia-Ochoa and Gomez, 2009). With the continuous maturity of related technologies, many bioreactors with low shear force have been designed to overcome the effects of shear forces generated by stirred bioreactors on animal cells (Mazzei et al., 2010; Ding et al., 2019; Gharravi, 2019).

The key to the large-scale industrialization and commercialization of cell culture technology lies in designing a suitable bioreactor (Nienow, 2006). The cell culture reactor is also the key apparatus in the whole vaccine production process. It provides a suitable growth environment for cells, determines the quality and yield of cultured cells, and affects the vaccine’s production efficiency and product quality (Gallo-Ramírez et al., 2015; Tapia et al., 2016). Virus vaccine production can also use a continuous multistage culture system (Chang et al., 2014; Tapia et al., 2016) or a solidifier (Kilburn and van Wezel, 1970). As two-stage bioreactors (Frensing et al., 2013), continuous bioreactors can be operated under steady-state conditions (fixed cell and metabolite concentrations, and pH, and avoiding a shutdown for cleaning and sterilization). Cell culture bioreactors can be divided into mechanical stirring bioreactors (YekrangSafakar et al., 2018; Schirmer et al., 2021), airlift bioreactors (Al-Mashhadani et al., 2015), hollow fiber bioreactors (Knazek et al., 1972; Jyothilekshmi and Jayaprakash, 2021), and disposable bioreactors (Junne and Neubauer, 2018; Jyothilekshmi and Jayaprakash, 2021). According to how the cells are cultured, these bioreactors can be divided into three types: Adherent culture bioreactors, embedded culture bioreactors, and suspension culture bioreactor. We have a list description of the advantages and disadvantages of these bioreactors, and the biosafety risks associated with bioreactors (Table 1).

**TABLE 1:** Types of cell culture bioreactors and biosafety risks.

Bioreactor type	Main form	Advantages	Disadvantages	Biosafety risks
Adherent culture (Atlas, 1999; Al-Mashhadani et al., 2015; YekrangSafakar et al., 2018; Schirmer et al., 2021)	Stirring bioreactor	• Simple and flexible operation	• Need for high cell numbers	• Small bioreactors can create substantial amounts of biological weapons with relative ease (Stacey et al., 2012).
	Hollow fiber bioreactor	• Saving time		• If infinite cell lines are utilized, there may be a risk of tumorigenesis (Bou and De Wilde, 2014).
	Torrent pouring bioreactor	• pH, dissolved oxygen and temperature can be monitored online by dielectric constant sensor	• Difficulty to scale-up processes	• Operators may make more mistakes when implementing the large-scale batch culture than in small scale (Pollard and Pralong, 2018).
Embedded culture (Kumar and Shuler, 1995; Singh, 1999; Valkama et al., 2018)	Fluidized bed bioreactor	• Minimize the damage to cells caused by the shear force	• Difficulty to achieve sufficient dissolved oxygen in large-scale culture	• Without proper testing in single-use bioreactors, high temperature, high pressure, high friction, and sharp objects might damage the bag, resulting in liquid or gas leakage and operator contamination (Wang et al., 2015).
		• Easy for cells to culture and grow
	Fixed bed bioreactor	• Prolong the survival time of cells after infection	• Hard for scale-up	
Suspension culture (Merchuk, 2003; Rourou et al., 2019; Wu et al., 2021; Zhang et al., 2021)	Stirring bioreactor	• Suitable for CHO cells to produce recombinant proteins or insect cells for baculovirus expression systems to produce virus-like particles	• High liquid shear force and low actual utilization rate	
		• Easy to expand the scale of cultivation	• Long construction period	
		• Achieve a relatively uniform microenvironment	• Poor operational flexibility	
	Airlift bioreactor	• Condition parameters are relatively controllable and stable	• High one-time investment costs	

In recent years, static cell line equipment of all sizes has been updated and improved (Tapia et al., 2016). Currently, disposable fixed-bed bioreactors, such as the iCELLis ^®^500 hand 500^+^ system, have been used in adherent cell culture techniques to produce high yields of live viruses (Leinonen et al., 2019; Valkama et al., 2020). The operation of the disposable bed bioreactor is simple and flexible, which saves time, and the culture parameters, such as pH, dissolved oxygen (DO), and temperature, can be monitored online using a dielectric constant sensor. In the production of live recombinant vesicular stomatitis virus vaccine based on Vero cell, some studies used the latest Univercells’s scale-X fixed-bed bioreactor system, with an increase of 2–4 virus titers per surface area (Berrie et al., 2020). The latest invention in the fixed-bed bioreactor area is Corning’s Ascent Bioreactor, which uses different low-shear strategies to provide oxygen and nutrients to cells (Tara and Hannah, 2001). Enzymatic digestion can separate cells in 2D culture, making it ideal for high-yield and scable viral vector synthesis (Freshney, 2015). For the application of *in vitro* gene therapy, because of a lack of medium-sized bioreactors in the iCELLis series, which might be more suitable for *in vitro* therapy, Univercells has launched the competitive fixed-bed bioreactor scale-X biological series (Leinonen, 2022). Studies have shown that lentivirus and adenovirus vectors have the same productivity in iCELLisNano and scale-XHydro bioreactors, but the distribution of cells in the scale-XHydro is better than that in the iCELLisNano (Leinonen, 2022). In the adherent cell culture mode, the cells can only adhere to the rotating bottle wall, and if the cell growth needs to be magnified, it will be limited by manpower, cost, and plant space; therefore, it is difficult to achieve process magnification (Emeny and Morgan, 1979). These limitations have promoted the research progress of suspension culture. For process optimization, the choice of suspension culture can be considered better than the adherent process (Schlaeger and Christensen, 1999). For example, Cytiva’s newly developed Wave 25 bioreactor has advanced sensors, intelligent control strategies, and other functions to enhance rocking technology, resulting in reliable and accurate performance, in terms of maintaining cell activity and increasing virus production (Zhang et al., 2021). The Ambr250 bioreactor developed by Sartorius can run up to 48 parallel bioreactors with a culture volume of 100–250 ml. It has a small-scale multi-module system, which is a defect of adherent bioreactors (Rotondi et al., 2021). The advantage of suspension culture is to achieve large-scale production with more advanced automation technology. In future large-scale production, suspension culture will inevitably replace the traditional adherent culture and become the mainstream method.

### 3.2 Increasing virus yield through process optimization

**FIGURE 2:**
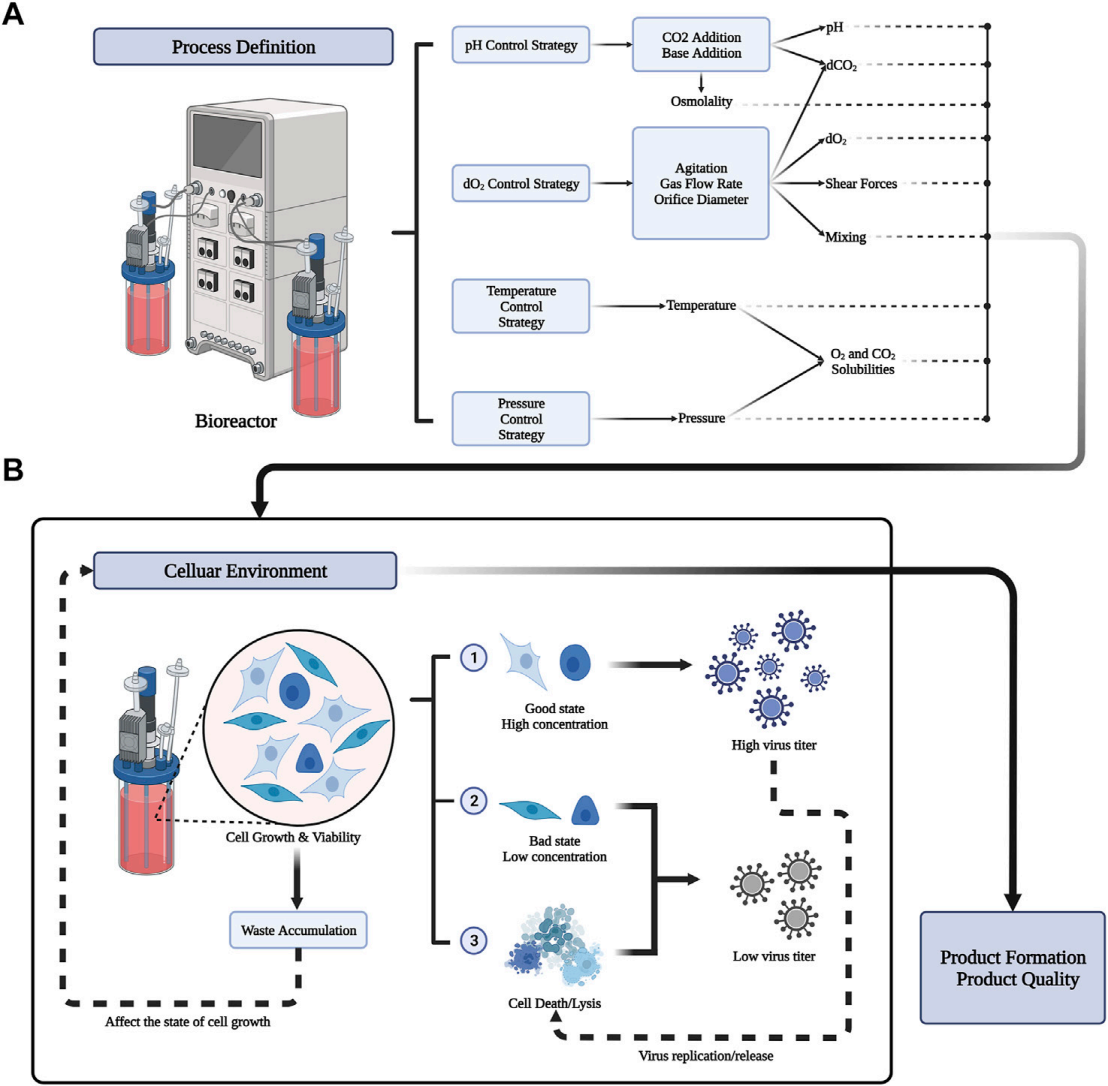
Process definition in bioreactor and its result influence in celluar environment. **(A)** Bioreactors can preset important parameter values through four strategies (pH Control Srategy, dO2 Control Strategy, Temperature Control strategy, pressure control strategy). The output of the process includes physical and chemical parameters such as pH, solubility of different gases, temperature, osmotic pressure, shear force and so on. Different process definitions have a great influence on the cellular environment. **(B)** Different celluar environment will lead to different growth states of cells. Good cell state and higher cell concentration can produce more virus titers. But the replication and release of the virus can also lead to cell death. Meanwhile, the accumulation of waste from cell metabolism will affect the celluar environment. The state of the cellular environment ultimately determines the formation and quality of the product.

The purpose of upstream biological process development is to reduce the overall manufacturing cost. In principle, the more cell lines that act as substrates, the greater the amount of virus produced (Gallo-Ramírez et al., 2015; Genzel, 2015; Tapia et al., 2016; Kiesslich and Kamen, 2020). In addition, keeping the cell line in its optimal state is another key factor to produce a high amount of virus (Freshney, 2015; Tapia et al., 2016; Kiesslich and Kamen, 2020). It is important to maintain the cell line in optimal conditions for viral production throughout upstream process, such that as many cell materials as possible can be produced. This might be achieved by adjusting several parameters throughout the manufacturing process. The process definition of different parameters in the bioreactor is shown in Figure 2A. The process parameters include physical and chemical parameters (temperature, pH, DO, osmotic pressure, shear force, and nutrient supply) (Tapia et al., 2016; Kiesslich and Kamen, 2020), the virus multiplicity of infection (MOI), infection time (TOI), organic nutrients, and inorganic ions. In the following sections, we will introduce the above parameters and the current mainstream cell culture methods.

#### 3.2.1 Cell concentration and metabolic/physiological status

In the stage of cell proliferation, most of the biological products produced by animal cell culture are continuously replicated and synthesized. For example, recombinant proteins are usually produced in batches or in batches, and can be harvested once the concentration reaches a peak in the culture medium (Castilho et al., 2008). By contrast, the production of viral vaccines usually requires a stage of cell growth. Through the virus replication phase (batch mode), most viruses replicate continuously through multiple infections in a complex process. This includes the synthesis of host cells, viral RNA/DNA and viral proteins, and the release of offspring viruses (Aunins, 2003). Viral replication and release usually lead to cell lysis, such that viral materials can be harvested at their peak concentration in batches. As a general rule, the cell concentration determines the final virus titer. The key nutrients of cell culture and cumulative by-products can inhibit virus amplification, such as lactic acid and ammonia (DeMarchi and Kaplan, 1977; Park et al., 2021). The effect of cell state on virus production in the cellular environment is shown in Figure 2B. Reasonable parameters can be simulated in small-scale culture to provide an optimal cellular environment. However, optimize parameters in large-scale culture will pay a higher price.

#### 3.2.2 Shear stress

The process of parallel layers sliding past each other is known as shearing. Shear forces from agitation and sparging might impact enveloped virus generation in bioreactors (Grein et al., 2019). For example, Grein et al. (2019) discovered that the measles virus is vulnerable to shear stress in bioreactors. They found that in several cases, agitation and sparging lowered the virus titer by 1000-fold. In addition, headspace aeration can provide enough oxygen for cell culture to reduce the shear effect caused by spraying (Betts et al., 2014). Currently, there are new bioreactors that can overcome the influence of shear stress to a great extent. Sartorius Stedim Biotech’s disposable bioreactor BIOSTAT ®RMTX is equipped with a disposable Flexsafe ®RMTX process bag for mild stirring, which is designed with special ports for non-manual gravity harvesting (Hupfeld et al., 2020). And this unique gravity harvest concept avoids the risk of contamination caused by manual operation, reduces the effect of shear stress on fragile cells, and maximizes the recovery of cells (Hupfeld et al., 2020). Although shear forces are much lower in adherent culture, it is difficult to scale up. Suspension culture is the exact oppsite. Therefore, methods for reducing shear stress in suspension culture should be further explored to improve virus yield in large-scale culture.

#### 3.2.3 Multiplicity of infection

The virus mainly spreads to the target cell in the culture medium; therefore, the degradation/inactivation of virus before it reaches the target cell should be considered when calculating the optimal inoculation number of the virus (Tapia et al., 2016). Secondary infection is a crucial factor in continuous culture. In this process, the transport and spread of virus and re-adsorption infection follows a complex mathematical model (Nielsen, 2003). In the case of cascade amplification of the stirring tank bioreactor to culture the virus, there is generally an optimal MOI value, i.e., the highest titer of the virus that can be harvested. In addition, for most viruses, if the amount of virus particles per unit cell is too high at the time of infection, this might lead to the replication of so-called defect interference particles, reducing the maximum virus production that can be achieved (Frensing, 2015).

#### 3.2.4 Residence time and the time point of harvest

When the cell or virus is retained in a closed system it runs in batch culture mode. In general, when the maximum titer is reached, the infectious titer of the virus and the total number of virus particles will start to decrease (Nielsen, 2003). However, the point when the pollution level of extracellular DNA and protein start to increase significantly is the most suitable time to harvest the virus.

Unlike the classical recombinant protein production process, during the viral vaccine production process, the parameter conditions used are different during the cell growth and viral replication phases. Therefore, specific process strategies for different stages must be used (Tapia et al., 2016). Under steady-state conditions, to achieve a relatively high production capacity and low cost for viral vaccine production, it is necessary to determine the best virus retention time and harvest time (Jordan et al., 2013). This can prevent the rapid degradation of the virus and associated yield reduction, and the proportion of infectious virus particles will be relatively high, which is beneficial for the production of live attenuated vaccines or viral vectors, such as recombinant vaccinia vaccine (Jordan et al., 2020) and influenza A virus vaccine (Walther et al., 2015). However, it is unclear whether process control and monitoring can be carried out in viral vaccine production. Several open questions raised by the Food and Drug Administration and European Medicines Agency about the long-term genetic stability of cells and virus strains remain to be answered (Gallo-Ramírez et al., 2015), especially whether virus mutations will occur caused by overproduction in the process of continuous culture and the negative effects of time on product efficiency and safety.

#### 3.2.5 Microcarrier culture

Microcarrier culture is recognized as the most promising technique for large-scale culture of animal cells, which has the advantages of both suspension culture and adherent culture (Blüml, 2007). The harmLess microcarrier particles are added to the culture medium in the culture container to allow the cells to attach and grow on the surface of the microcarrier and the microcarriers are kept in suspension by continuous stirring (van Wezel, 1967).

Adherent cells show differences in cell density because the difference in the maximum available growth area. Microcarriers are often added to the bioreactor to increase the cell density of adherent cells (Genzel et al., 2010; YekrangSafakar et al., 2018; Rourou et al., 2019). Rourou et al. showed that Vero cells grown on 3 g/L of the microcarrier Cytodex 1 could obtain a cell density level of 2.6 × 10^6^ cells/mL (Rourou et al., 2019). However, one of the disadvantages of microcarriers is that many cells are required to be inoculated, which might aggravate the cell density effect and make it difficult to expand the production scale (Gallo-Ramírez et al., 2015). To produce and maintain many adhesion-dependent cells, stirring tank bioreactors based on microcarriers are usually used; however, this can cause a cytopathic effect (CPE) by harmful hydrodynamic shear stress in the bioreactor (YekrangSafakar et al., 2018). As a new solution, hollow microcarriers (HMCs) have been proposed to protect cells from shear stress in stirred bioreactors. Meanwhile, it also can ensure adequate gas and nutrients, and uniform mass transfer rates. This is conducive to the large-scale expansion of shear-sensitive anchoring-dependent cells on an industrial scale (YekrangSafakar et al., 2018).

#### 3.2.6 High cell density virus culture

With the increasing demand for vaccines, the production process of virus vaccines based on cell culture also requires a series of technological enhancements to overcome the shortcomings of traditional vaccine production. For many conventional cell lines used in vaccine production, the number of producing cells ranges from 2 × 10^6^ cells/mL to 4 × 10^6^ cells/mL, which is a high cell density (Riesenberg and Guthke, 1999). Some processes and methods for high cell density processes have been developed, and many bioreactor products (Acoustic filters, Hollow-fiber based system and CellTank^®^ etc.) have entered the market (Tapia et al., 2016; Granicher et al., 2019). It has been found that through improved culture strategies, such as fed-batch (Gutiérrez-Granados et al., 2018) and perfusion strategies (Teworte et al., 2021), high cell density can be obtained. However, high cell density culture will decrease cell-specific virus production and appear the cell density effect (i.e., the drop in the specific productivity in the virus-cells expression system when cells are infected at high cell densities). The cell density effect is a widespread phenomenon (Bernal et al., 2009; Clincke et al., 2013). Studies have shown that the cell density effect can be limited by perfusion technology, and the accumulation of unnecessary by-products can be avoided by providing a continuous nutrient-rich environment, thus maintaining cell-specific yield (Gutiérrez-Granados et al., 2018; Schmid et al., 2018; Nikolay et al., 2020a).

The culture volume of perfusion culture is small, the recovery volume is large, and the product stays in the tank for a short time; therefore, it can be recovered and preserved at low temperature in a short time, which is conducive to maintaining the activity of the product (Nikolay et al., 2020a). However, the operation of perfusion culture is complicated, and the utilization efficiency of cell medium is low (Tapia et al., 2016). Therefore, it might be necessary to change the medium in time in the culture (Schmid et al., 2018; Nikolay et al., 2020b). A study showed that after obtaining the optimum ratio and adjusting the concentration of some nutrients and the osmotic pressure, the obtained medium needs only half the perfusion rate to maintain a density of 3 × 10^7^ cells/mL (Lin et al., 2017). This provides a reference for the development of perfusion medium. High density and high viability are the major features of perfusion culture; however, it is impossible to increase cell density indefinitely. Too high cell density will make the medium viscosity too high such that subsequent culture can’t be carried out (Bernal et al., 2009; Clincke et al., 2013). To control the cell density at a stable level, it is necessary to adjust the perfusion rate. Recent studies have shown that through the detection of the cell-specific perfusion rate and substrate metabolism, the perfusion rate can be automated and controlled with high precision to achieve a higher cell concentration, and the virus concentration can reach 1 × 10^10^ virus/mL (Nikolay et al., 2020a). However, the operation of perfusion culture is complicated and the utilization efficiency of cell medium is low. For perfusion culture technology, the process of medium replacement might lead to an increase in the risk of asepsis, and the system cannot be operated with the best medium exchange rate (Nikolay et al., 2018a; Granicher et al., 2019; Nikolay et al., 2020a).

## 4 Large-scale cultures of different types of viruses

Process optimization includes the optimization of cell lines, physical and chemical parameters, microcarrier culture technology, and high cell density culture technology. When the same virus grows in different cell lines, culture media, and bioreactors, the virus production can vary, and only changing the temperature, pH, MOI, and other physical and chemical conditions while keeping the other conditions the same can also have a significant effect on virus yield (Frazzati-Gallina et al., 2001; Trabelsi et al., 2005; Tapia et al., 2016). Therefore, finding the optimal conditions suitable for the growth of different viruses will help to greatly increase virus production, thus increasing vaccine production and meet the global vaccine demand. In this section, we will summarize and comment on the specific process optimization methods for the large-scale production of SARS-CoV-2, influenza virus, tropical virus, enterovirus, and rabies virus (Table 2).

**TABLE 2:** Overview of virus production process in the cell culture reported in the review.

Virus	Bioreactor type	Cell line	Cell culture Methods/Culture medium	Microcarrier	Physical and chemical parameters	Optimum inoculation density	Virus highest yields	Comments	Refs.
SARS-CoV-2	CelCradle TM500-AP	Vero	animal-free medium	BioNOC ™II	At 72 hpi, MOI = 0.006, 33 °C	(2.2–2.5) × 10^9^ cells/vial	7.3 log10 TCID_50_/mL	Virus generation in the CelCradle TM 500-AP was more efficient than in monolayer cells	Offersgaard et al. (2021)
SARS-CoV-2	Basket reactor	Vero	—	No	After 48–72 h of infection, the MOI ranged from 0.01 to 0.3	—	7.0 log10 CCID_50_/mL	The solid-flow bed technology makes high-density cell culturing easier	Wang et al. (2020b)
SARS-CoV-2	Pall Allegro™	HEK293	BalanCDHEK293 medium	No	At 42–48 hpi, the MOI ranged from 5 to 10	(2–3) × 10^6^ cells/mL	5 × 10^11^ VP/mL	This production efficiency is double that of the prior batch or feed batch	Joe et al. (2022)
SARS-CoV-2	Perfusion bioreactor	—	perfusion	No	MOI = 70	—	1 × 10^12^ VP/mL	Perfusion culture yields 10 times more than batch culture	Trabelsi et al. (2019)
rVSV_Ind_-msp-sf-G_tc_ (SARS-CoV-2)	Stirring tank bioreactor	Vero	MDXK medium	No	At 48 hpi, MOI = 0.01, 31°C, pH = 7.2, DO50%	1.02 × 10^6^ cells/mL	3.59 × 10^9^ TCID50/ml (Infectious titer)2.13 × 10^10^ VG/mL (Genomic titer)	In MDXK, total viral particles to infected particle is 3.0VG/TCID50	Kiesslich et al. (2021)
H1N1	Perfusion bioreactor	MDCK	semi-perfusion, Smif8 medium	No	At 30 hpi, MOI = 0.1, 37°C	6 × 10^7^ cells/mL	4.5 log10 (HAU/100 ml)1 × 10^10^ TCID50CSVY = 13600 virions/cell	Semi-perfusion let the virus grow and infect MDCK.Xeno cell line in a high cell density environment	Bissinger et al. (2019)
A/PR/8/34 H1N1	Stirring tank bioreactor	MDCK	perfusion, Xeno-CDM2	No	pH = 7.15 (cell growth), pH = 7.20 (virus infection), DO = 40%37°C (cell growth), 33°C (virus infection)CSPR = 40 pL/cells/day	45×10^6^ cells/mL	4.42 log10 (HAU/100 μL),C _tot_ = 5.3 × 10^11^ virions/mlC _tot, infectious_ = 18 × 10^9^ virions/mLCSVY = 11690 virions/cellSTVY = 8.0 × 10^13^ virions/L/d	STVY of improved high cell density method is 5 times traditional batch process	Wu et al. (2019)
A/PR/8/34 H1N1	DASGIP^®^ bioreactor	PBG.PK2.1	perfusion, CD-U5 medium	No	At 36 hpi, MOI = 10^–5^, 37 °C pH = 7.2 (cell growth), pH = 7.4 (virus infection)CSPR = 0.07 nL/cell/day	5 × 10^6^ cells/mL	46 × 10^6^ TCID50/mL3.93 ± 0.05 log10 (HA units/100 ml)	Use the PBG.PK.2.1 Cell yielded higher TCID50. However, the CSVY was still smaller than the MDCK cells	Granicher et al. (2019)
H1N1	SB10- X orbital shaking bioreactor (OSB)	AGE1.CR.pIX	perfusion, CD-U3 medium	No	37°C	5 × 10^7^ cells/mL	3.73 log10 (HA unit/100 ml)CSVY = 3,500 virions/cellPv = 2.2 × 10^12^ virions/L/d, 8.8 ± 10^9^ TCID50/mL	OSB is more useful in increasing the CSVY and Pv of AGE1.CR.pIX cells than other bioreactors	Coronel et al. (2020)
A/PR/8/34 H1N1	Inclined settler (IS)	AGE1.CR.pIX	perfusion, CD-U3 medium	No	At 36–48 hpi, 27°CCSPR = 0.06 nL/cell/day	5 × 10^7^ cells/mL	25 × 10^6^ TCID50/mL CSVY = 3,474 virions/cell Vir _tot, max_ = 6.5 × 10^13^ virions Pv = 1.23 × 10^12^ virions/L/d	Using IS, the yield of cell-specific virus was approximately 5 times higher than that of the ATF basal culture.	Liu et al. (2008)
Enterovirus 71	Disposable perfusion bioreactor	Vero	perfusion, DMEM	No	At 96 hpi, MOI = 0.1, 32 °C	1.0 × 10^7^ cells/vial	8.0 log10 TCID_50_/mL	The bioreactor provides a high oxygen transfer efficiency, which makes it very suitable for virus culture	Liu et al. (2018)
Enterovirus 71	BIOFLO 310 bioreactor	Vero	perfusion, serum-free VP-SFM medium	Cytodex 1	At 7 to 13 dpi, MOI = 10^–5^, 32 °C	(2.0–2.5) × 10^6^ cells/mL	1.0 × 10^7^ TCID_50_/mL	The medium replacement culture strategy was found to increase the production yields more than 7–14 fold	Wu et al. (2015)
Inactivated EV71 (E59-B4) virus	Serum-Free Microcarrier Bioreactor System	Vero	serum-free VP-SFM medium	Cytodex 1	At 6 days, MOI = 10^–4^, 20 rpm, pH = 6.8–7.2, 37°C	1.0 × 10^6^ cells/mL	1.0 × 10^7^ TCID_50_/mL	Microcarrier/bioreactor is more efficient than rolling bottle system	Chen et al. (2021)

Virus	Bioreactor type	Cell line	Culture medium	Microcarrier	Physical and chemical parameters	Optimum inoculation density	Virus highest yields	Comments	Refs.
Coxsackie virus A16	AmProtein Current perfusion bioreactor	Vero	Serum-free medium	No	At 5 days, MOI = 0.5, 33 °C	1.5 × 10^7^ cells/0.6 g plate	8.0 log10 TCID_50_/mL	Using fiber paper carrier to amplify virus; Novel disposable perfusion bioreactor	Nikolay et al. (2018b)
Zika virus	Mobius^®^ Single-use Bioreactor	BHK-21SUS	Bovine serum with DMEM	No	At 4 days, MOI = 0.001, pH = 7.1	1.2 × 10^7^ cells/mL	3.9 × 10^7^ PFU/ml	Total virus particles increase, but cell-specific production remained lower than in adherent cell lines	Nikolay et al. (2018a)
Zika virus	Perfusion bioreactor	EB66^®^ cells	GMEM with 10% FBS	No	At 2 days, MOI = 0.001, 34 °C or 37 °C	1.6 × 10^8^ cells/mL	1.0 × 10^10^ PFU/ml	The enhancement of EB66 ®cell culture process significantly increased the production of Zika virus	Rourou et al. (2007)
Dengue virus	Bellco spinner flasks	Vero	M-VSFM medium	Cytodex 1	MOI = 0.01	6 × 10^5^ cells/mL	2.2 × 10^7^ PFU/ml	The peak titer of virus produced by Vero cells was about 1–17 times higher than that of MRC-5 cells.	Lee et al. (2011)
Chikungunya virus	DASGIP^®^ bioreactor	Sf9s cells	Sf-900 II SFM medium	No	At 52 hpi, MOI = 0.01 TCID50/cell 27°C, pH = 6.3	2 × 10^6^ cells/mL	chikv VLP = 2.1 mg/L	DASGIP Bioblock could control the temperature more efficiently	Rajendran et al. (2014)
Chikungunya virus	iCELLis Nano bioreactor	Vero	Minimum essential medium	No	—	4 × 106 cells/mL	1.4 × 10^9^ pfu/ml	Chikungunya virus titers from iCELLis Nano bioreactor was twice higher than Commercial packedbed system and Roller culture bottles	Tomori and Kolawole, (2021)
Rabies	Stirring tank bioreactor	Vero	VP-SFM medium	Cytodex 1	At 3 days, MOI = 0.1, 34°C, pH = 7.4	5 × 10^6^ cells/mL	1.38 × 10^8^ FFU/mL	Mixture of inactivated viruses had the same titer as serum-supplemented medium	Kiesslich et al. (2020b)
LP-2061 Rabies	Stirring tank bioreactor	Vero	VP-SFM medium	Cytodex 1	At 10 days, MOI = 0.05, 37°C, pH = 7.4	3.88 × 10^6^ cells/mL	4.8 × 10^7^ FFU/mL	In VP-SFM medium, suspension culture produced more virus-specific productivity than adherent culture	Atlas, (1999)
LP-2061 Rabies	Shake flasks	Vero	IPT-AFM medium	Cytodex 1	MOI = 0.1, 37°C, 5% CO_2_	8 ± 0.5 × 10^5^ cells/mL	5.2 ± 0.5 × 10^7^ FFU/mL	IPT-AFM medium with reduced Ca^++^ and Mg^++^ content increased rabies virus production	Rourou et al. (2019)
Ebola virus	Single-use scale-X™ hydro fixed-bed	Vero	VP-SFM medium	Cytodex 1	At 24 hpi, MOI = 0.01, 34°C, DO50%	2.7 × 10^5^ cells/cm^2^	1.95 × 10^7^ TCID50/mL 101VG/TCID50	Cell-specific infectious particle production increased 1.9 times, but total virion production per cell decreased 5.9 times.	Kiesslich et al. (2020b)
rVSV-ZEBOV	Stirring tank bioreactor	Vero	MDXK medium	No	At 24 hpi, MOI = 0.01, 34°C, pH = 7.2, DO50%	1.02 × 10^6^ cells/mL	3.87 × 10^7^ TCID50/mL (Infectious titer) 1.09 × 10^10^ VG/m (Genomic titer)	The ratio of total viral particles to infectious particles in MDXK was 282 VG/TCID_50_	Kiesslich et al. (2021)

hpi, hours post-infection; TCID50, 50% tissue culture infective dose; CCID50, 50% cell culture infective dose; VP, viral particles; VG, viral genomes; HAU, hemagglutination units; HA, hemagglutinin; CSVY, cell-specific virus yield; STVY, space time virus yield; Ctot, the total number of virus particles per volume; CSPR, cell-specific perfusion rate; Pv, volumetric virus productivity; Vir tot, max, maximum total number of virions produced; PFU, plaque forming unit; ATF, alternating tangential flow; TFF, tangential flow filtration; DMEM, Dulbecco’s modified Eagle’s medium; FBS, fetal bovine serum; SFM; serum free medium; MOI, multiplicity of infection; DO50%, 50% dissolved oxygen; DO, dissolved oxygen; rpm, revolutions per minute; ACPB, AmProtein Current Perfusion Bioreactor; GMEM, Glasgow minimum essential medium; M-VSFM, modified Vero serum-free medium; VP-SFM, virus particle-serum free medium; DMSO, dimethyl sulfoxide; IPT-AFM, animal-component free medium; MDXK, chemically-defined and developed by Xell for cultivation of MDCK/MDBK and other mammalin cell lines; FFU, focus forming units; EILV, Eilat virus.

### 4.1 SARS-CoV-2

In 2020, the COVID-19 pandemic broke out globally, which posed a huge threat to public safety. The World Health Organization (WHO) has declared a public health emergency (Gao et al., 2020b; Wang et al., 2020c; Li et al., 2020). Given the severity of the COVID-19 pandemic, it has become a research hotspot to develop a safe and effective vaccine against SARS-CoV-2 infection. At present, there are 195 vaccine candidates in the preclinical development stage and 144 vaccine candidates in the clinical development stage (as of 20 February 2022) (Basta and Moodie, 2020; World Health Organization, 2022b). They mainly use four vaccine platforms: inactivated virus, protein subunit, adenovirus vector, and mRNA. In the face of the increasingly tense pandemic situation, it is urgent to use more efficient biological processes to produce more vaccines to meet the global vaccine demand.

There have been a few studies describing the large-scale amplification of SARS-CoV-2 in bioreactors. For example, Offersgaard’s team (Offersgaard et al., 2021) inoculated 1.5 × 10^8^ Vero cells in a 0.5 L CelCradle TM500-AP vial with a 5.5 g BioNOC ™II carrier, and after 7 days of planting in an animal-free medium, the total number of cells reached (2.2–2.5) × 10^9^ cells/vial. When the MOI was 0.006, and the virus temperature was 33°C, the peak titer of SARS-CoV-2 infection was 7.3 log10 50% tissue culture infective dose (TCID50)/mL after 72 h of infection and the titer of six harvests was ≥6.5 log10 TCID50/mL. A total of 10.5 log_10_ TCID_50_ was produced (about 5 L).

BBIBP-CorV vaccine is the first SARS-CoV-2 inactivated vaccine in the world (Xia et al., 2021). In contrast to the CelCradle TM500-AP, a BBIBP-CorV inventory production strategy based on a novel vector in a basket reactor has been developed to ensure efficient production (Wang et al., 2020b). The MOI ranged from 0.01 to 0.3 and after 48–72 h of infection, they obtained 7.0 log10 50% cell culture infective dose (CCID_50_)/mL. In the solid-flow bed culture system, the cultured cells are in a relatively static state, which makes the difficult high-density cell culture simple and easy. Apart from these, according to Wang et al., the HB02 strain produced the highest virus yield in Vero cells among three candidate virus strains and showed no amino acid variation within 10 generations, which means that the various types of virus strain will greatly influence final production. (Wang et al., 2020b).

ChAdOx1n CoV-19 (AZD1222, Vaxzevria) is an effective adenovirus vector-based vaccine against SARS-CoV-2 (van Doremalen et al., 2020). The virus used to make the vaccine can be produced by HEK293 cells. Joe et al. (2022) cultured HEK293 cells in BalanCDHEK293 medium and a Pall Allegro bioreactor. Finally, the cells grow to about (van Wezel, 1967; Griffiths, 1990) × 10^6^ cells/m. And when the MOI ranged from 5 to 10, and at 42–48 h post infection (hpi) and the adenovirus vector reached to 5 × 10^11^ virus particle (VP)/mL. The team successfully achieved the largest viral vector manufacturing activity to date, providing a significant proportion of the global COVID-19 vaccine supply at low cost. This production efficiency is approximately twice that of previously disclosed batch production or feed-in batch production of adenovirus. Therefore, perfusion culture is suitable not only for ordinary mammalian cell lines, but also for the production of adenovirus vectors.

Research on a SARS-CoV-2 vaccine based on rVSV vector is also ongoing (Henao-Restrepo et al., 2015). Kiesslich et al. compared the production of rVSVInd-msp-sf-Gtc in MDXK medium and IHM03 medium respectively (Kiesslich et al., 2021). They grew Vero cells in MDXK medium and provided 31°C, pH 7.2, dissolved oxygen (DO) in a 50% culture environment to achieve an optimal cell density of 1.02 × 106 cells/mL. At 48hpi, the MOI was 0.01, the infection titer was 3.59 × 10^9^TCID50/mL, and genomic titer was 2.13 × 1010 viral genome (VG)/mL (Kiesslich et al., 2021).

The current COVID-19 vaccines developed using recombinant protein technology are on the market [such as ZF 2001 (An et al., 2022) and NVX-CoV2373 (Tian et al., 2021)]. Antigenic genes are the focus of this vaccine, not the entire virus. And it also requires CHO cells, many of which have been genetically engineered for transient expression, such as Epi CHO, CHO Freestyle Max™, and CHO-S (Geisse and Voedisch, 2012). Johari et al. (2021) placed CHO-S cells in suspension culture in chemically-defined medium optimized for the growth of CHO cells (CDCHO) medium containing 8 mM L-glutamine, and the temperature was kept at 37 °C. When CHO-S cells density reached 1.5 × 10^6^ cells/mL, they were treated with polyethyleneimine (PEI). Then, the vector with a 100 relative promoter units (RPU) promoter and the spike gene was transfected into the electroporated cells, and the temperature was reduced to 32 °C for fed-batch manufacturing. Finally, 53 mg/L of purified spike protein was harvested. Similarly, Pino et al. (2020) suspended codon optimized CHO Express™ cells in EX-CELL^®^ Advanced™ CHO medium and maintained a temperature of 37°C, before retransfection with vector pXLG6 (ExcellGene SA) containing SARS-CoV-2 Spike DNA sequences. After 10 days, it was transferred to medium feed production in 50 ml Tube Spin^®^ bioreactor (TPP, Trasadingen, Switzerland) and cooled to 31 °C. This culture was then inoculated into 10 and 40 L stirring tank bioreactor at 5 × 10^5^ cells/mL, resulting in a significant yield increase (data not listed). Therefore, the CHO expression system not only results in high yield but also can be used in a large-scale bioreactor.

Vero cells are widely used in the production of SARS-COV-2 because of their high sensitivity, and CHO cells expressing viral proteins are also the focus of current research (Geisse and Voedisch, 2012; Pino et al., 2020; An et al., 2022). In contrast to the production of other viruses, there are more brand new bioreactors (Offersgaard et al., 2021; Joe et al., 2022) for the production of SARS-COV-2. However, it is still necessary to strengthen the environmental monitoring throughout the culture process and optimize the culture medium.

### 4.2 Influenza virus

Similar to SARS-CoV-2, influenza A virus also poses a significant threat to world health. For the treatment of influenza diseases, vaccines are the most effective and safe method (Kostova et al., 2013). A process based on cell culture has also been established. Different host cell lines, such as MDCK, Vero, AGE1.CR, or PER.C6 cells can be used to produce influenza viruses. For example, Lai et al. used a new mammalian cell line, PBG.PK2.1 and hollow fiber ATF system (ATF2) high-density culture. Finally, the cell concentration was as high as 50 × 10^6^ cells/mL and the maximum HA titer was 3.93 log_10_ (HA unit/100 ml). PBG.PK2.1 cells can reach high cell concentration. The chemically defined medium can be used in cell culture and can be magnified on a simple scale, so it is a very promising candidate cell line for vaccine production. In addition, they also used the reactor for two perfusion cultures, the infection concentration of the cells reached about 4.6 × 10^6^/ml, and the maximum titer reached (3.93 ± 0.05) log_10_ (HA units/100 ml) (Gränicher et al., 2019). This confirms that use of perfusion devices can achieve better production and enables the continuous collection viruses. Therefore, more and more research tends to utilize perfusion technology. Bissinger et al. used MDCK cells in suspension culture under a semi-perfusion mode. When the MOI was 10^–1^, the virus titer could reach 4.5 log_10_ (HAU/100 ml), and the TCID_50_ could reach 10^10^ virions/mL (Bissinger et al., 2019). In another similar study from the same group, semi-perfusion culture was extended to a bioreactor with and ATF perfusion system. The team adjusted pH and temperature to achieve the accumulated HA titer (HA_aac_) value of 4.37 log_10_ (HAU/100 μL) (4.7 × 10^11^ virus/mL) (Coronel et al., 2019).

As mentioned above, the ATF perfusion system has some effect on virus production. Coronel et al. coupled an SB10-X orbital shaking bioreactor (OSB) for tangential flow filtration (TFF) and ATF. Under the optimal perfusion conditions of OSB, the CSVY could reach 3,500 virions/cells, the volumetric virus productivity (P_v_) was 2.2 × 10^12^ virions/L/d, the highest HA titer was 3.73 log_10_ (HA unit/100 ml), and the highest TCID_50_ titer was 8.8 × 10^9^ infectious virions/mL (115). But compared with the ATF system, they found tilt precipitator (inclined settler, IS) could get more virus. A total of (5.4–6.5) × 10^13^ virions were produced. When the trypsin activity was the highest in the IS (1.5 × 10^6^ U/cell or 38 U/mL), the cell population was completely infected in 24 hpi. When the infection concentration was 25 × 10^6^ cells/mL, the cell-specific virus production was as high as 3,474 virions per cell, significantly increasing IAV production (Liu et al., 2008). This brought influenza virus production to a new high. Therefore, the perfusion technology for influenza A virus production can be improved by introducing new perfusion bioreactors or adjusting physicochemical parameters in culture. Perfusion culture is easy to scale up, and there are heavy demand of influenza virus vaccines every year, so it is a good choice to use perfusion culture technology to expand the manufacture of influenza virus vaccines.

### 4.3 Tropical virus

Dengue fever is a mosquito-borne virus infection with four serotypes (DENV-1, DENV-2, DENV-3, DENV-4). Currently, only the Dengvaxia^®^ vaccine is licensed. Serum-free medium has been used for cell culture for many years. The studies of Liu CC et al. showed that the virus production of the four DEN serotypes observed in Vero cells or MRC-5 cells in serum-free medium was always 0.3 to 2.6 times higher than that in serum medium. And the peak virus titer in Vero cells was 1–17 times higher than that in MRC-5 cells (Lee et al., 2011). But the DEN-4 virus cloned from MRC-5 cells is more stable, as the Lee HC’s team confirmed in a follow-up study (Petersen et al., 2016). Although the gene stability of MRC-5 cells grown on microcarriers is stronger than that of Vero, its expanded culture is still a difficult problem.

Zika virus (ZIKV) belongs to the flavivirus genus of the flavivirus family and is mainly transmitted by mosquito bites (Nikolay et al., 2018b). Selection of cell lines and bioreactors also has a huge impact on Zika virus production. Nikolay et al. produced Zika virus from BHK-21 suspension cells grown in Dulbecco’s modified Eagle’s medium (DMEM), and used a 3 L disposable Mobius^®^ bioreactor with the pH maintained at 7.1. To improve the titer, the team used the ATF system to carry out perfusion culture. After 4 days of infection, the virus production reached 3.9 × 10^7^ PFU/ml when an MOI of 0.001 was used. Although the specific virus yield of suspension cells is still low compared with adherent cell culture, a large enough production process for ZIKV has been established (Nikolay et al., 2018a). With the progress of perfusion technology, the team also adopted hollow fiber-based perfusion processes in bioreactors equipped with an ATF/TFF system to optimize cell growth and increase virus titers. The virus was produced by EB66 ^®^ cells, which were cultured on Glasgow minimum essential medium (GMEM) at 34°C or 37°C, and at 2 days, the titer of ZIKV was 1.0 × 10^10^ PFU/ml when the MOI was 0.001. Its optimum inoculation cell density was 1.6 × 10^8^ cells/mL and the perfusion rate was controlled using a capacitive probe according to the cell concentration measured on-line, which successfully realized the automation of this process (Caglioti et al., 2013). Compared to before, the overall production went up by three orders of magnitude. This can be explained by the use of high cell density culture, EB66^®^ cell adaptive seed virus infection, and a hollow fiber-based perfusion system.

Chikungunya virus (CHIKV) is a mosquito-borne type A virus that has infected millions of people worldwide (Pijlman et al., 2020). At present, the virus has been successfully produced in insect cell lines. Pijlman et al. (Rajendran et al., 2014) optimized the DASGIP^®^ bioreactor and determined that the optimal MOI of BACe56-CHIKV recombinant virus was 0.01 TCID_50_, the optimum inoculation cell density was 2 × 10^6^ cells/mL, and the best harvest time was about 52 hpi. The concentration of CHIKV virus-like particle (VLP) was 2.1 mg/L. But another completely different study got Chikungunya virus from Vero cells, after cultivating 4×10^6^ cells/mL cells in the bioreactor, Ramya et al. (Tomori and Kolawole, 2021) seeded the virus into the cells and finally obtained a virus titer of 1.4 × 10^9^ PFU/ml. The current research focuses on develping the Chikungunya virus vaccine using recombinant viruses, and there are still few studies on the optimization process of bioreactor technology. But insect cells offer more options for the upstream process.

For Ebola virus, the most effective candidate vaccine is rVSV-ZEBOV (Gélinas et al., 2019). Gélinas et al. established a 3.5 L stirring tank bioreactor. HEK293SF cells were placed in HyClone HyCell TransFx-H medium at 34°C and infected at an MOI 0.001. When infected, the cell count was 1.16 × 10^6^ cells/mL and 1.8 × 10^6^ cells/mL at the end of production. The titer reached 1.19 × 10^8^ TCID_50_/mL and 50 TCID_50/_VG at 36 hpi. (Kiesslich et al., 2020a). However, Kiesslich et al. used the new fixed-bed bioreactor scale-X hydro to produce rVSV-ZEBOV at 34 °C and DO50%. Grown in VP-SFM medium, the maximum cell density was up to 271605 cells/cm2. When infected at an MOI = 0.01, the maximum infection titer was 1.95 × 10^7^ TCID_50_/mL at 24 hpi, and the ratio of virus genome to infection titer was only 101 VG/TCID_50_. Compared with the microcarrier system, the cell-specific productivity of infectious particles increased by 1.9 times, but the total virion productivity per cell decreased by 5.9 times (Wong et al., 2010). To improve the infection titer, they produced rVSV-ZEBOV in Vero cultured in SFM in IHM03 and MDXK medium, respectively, and cultured them in the same environment. Infected at MOI 0.01 in the IHM03 bioreactor, the infection titer was 1.05×10^7^ TCID_50_/mL, and the titer was 1.32 × 10^8^ TCID_50_/mL when the cell density was 4 × 10^8^ TCID_50_/mL. However, the suspension adapted Vero cells were more suitable for infection than MDCK, with a cell density of 1.02 × 10^6^ cells/mL, a maximum infection titer of 3.87 × 10^7^ TCID_50_/mL, a cell specific productivity was 37.9 TCID_50_/cell, and the ratio of total virus particles to infected particles of 282 VG/TCID_50_. Under similar culture environment conditions, with the improvement of the bioreactor, the yield and infection titer of rVSV-ZEBOV increased significantly (Kiesslich et al., 2021).

### 4.4 Enterovirus

Enterovirus 71 infections can cause hand, foot and mouth disease (HFMD) (Chong et al., 2014). The EV71 vaccine marketed in China in 2016 is used to prevent EV71-infected HFMD. Current EV71 vaccine candidates are produced from viruses cultured in Vero cells using a drum bottle or cell factory technology because they are easy to implement and operate (Wu et al., 2019). As the above technologies are labor intensive, a more efficient and economical process for producing EV71 is needed. Microcarrier bioreactor technology can provide a way for large-scale production and improve the potency of virus production. Chen et al. (Liu et al., 2018) used a novel micro bioreactor (Amprotein Inc., Zhejiang China) with a polymer fiber carrier. Under the conditions of an optimum MOI of 0.1 and an optimum temperature of 32°C, the maximum virus titer reached 1.0 × 108/ml at 3 days after infection, the total volume of the supernatant was 25 L, and the total yield of the virus was 1.93 × 1012. They created a new model structure and approach to culture for EV71 manufacture. Using this model system, a vaccine for HFMD might be created swiftly and affordably. Cytodex1 microcarriers, on the other hand, are widely used in the production of EV71. For example, Liu et al. (Wu et al., 2015) increased the yield of EV71 vaccine by microcarrier fusion bioreactor culture. The multi-harvested semi-batch (MHSBC) or perfusion cultures could significantly increase the EVA71 virus yield by 7–14 times compared with single batch culture. The results also showed that the perfusion technique has more advantages than the MHSBC culture method, and the culture medium replacement strategy can slow down the occurrence of a CPE and increase the yield of the EVA71 virus. Wu et al. (Chou et al., 2012) used a 200 L serum-free microcarrier bioreactor system to culture EV71 at 37°C, pH = 6.8–7.2, and an MOI = 10–4. The virus titer reached 107 TCID50/mL at 10 days after infection, and a large-scale vaccine platform for inactivated EV71 virus was established. These studies constitute valuable information on the development of a large-scale microcarrier cell culture process for producing inactivated EV71 vaccine. Meanwhile, the selection of culture medium is rate-limiting step for the final yield. In the pilot production of the EV71 candidate vaccine, it was found that the VP-SFM developed in the upstream process was the best medium for Vero cell growth and EV71 virus production (Cai et al., 2014).

Coxsackie A16 (CVA16) virus is also the main pathogen of HFMD, but there is no safe and effective preventive vaccine against CVA16 at present (Iwai et al., 2009; Chen et al., 2021). Further complicating the situation, EV71 virions do not elicit neutralizing antibodies that cross-react against CVA16 (Cai et al., 2014). Therefore, it is necessary to develop a candidate CVA16 vaccine based on Vero cell whole virus production. Based on the advantages of large-scale virus culture technology, Chen et al. (Steele and Fernandez, 2017) placed Vero cells on polymer fiber article carriers and provided a serum-free medium containing 0.5% (w/w) whey protein hydrolysates, using a disposable Bioflo310 and ammonia protein current perfusion bioreactor to monitor virus infection and Vero cell culture. After this optimization, the final virus titer reached 7.8 × 10^7^ TCID_50_/mL. This study showed that the low shear rate and friction force of fiber carrier can further reduce the cytopathic effect of virus on cells, so it is better than other microcarriers. Moreover, these researches will also promote the large-scale manufacture of inactivated CVA16 vaccines employing cell cultures grown on nonwoven polymer fiber paper.

### 4.5 Rabies virus

Rabies is a viral zoonosis. Dogs are the leading cause of human deaths from rabies, accounting for 99% of all rabies transmitted to humans (Hicks et al., 2012). Rabies vaccines derived from cell culture are still among the safest and most effective vaccines to prevent human rabies (Montagnon, 1989). Vero cells are widely used in rabies vaccine production (Rourou et al., 2007). The high-density cells in the bioreactor can continuously collect viruses through continuous perfusion culture. For example, Rourou et al. studied the perfusion culture of cell proliferation and virus proliferation in a 2 L bioreactor, and the cell density reached 5 × 10^6^/ml. The highest titer of the virus was 1.38 × 10^8^ focus forming units (FFU)/mL. The titer of mixed inactivated virus was 2.58 IU/ml (Nadal-Rey et al., 2021).

In recent years, Rourou further cultured rabies virus in suspension culture of Vero cells. The team established a serum-free culture system of Vero cells (VeroS) adapted to suspension in a shake flask with 5% CO_2_ at 37°C. Rabies virus LP-2061 strain was used to infect cells at an of MOI 0.1 and cell density (8 ± 0.5) ×10^5^ cells/mL. The virus titer of all tested media was higher than 10^7^ FFU/mL (Rourou et al., 2019). Compared with adherent culture, the virus-specific productivity of VeroS was slightly increased. This proves that the obtained VeroS is suitable for the production of high titer rabies virus and paves the way for developing a VeroS bioreactor process to produce rabies vaccine. However, the virus titer achieved by suspension culture with Vero is still smaller than that obtained by suspension. Therefore, the technology of Vero cell suspension culture is still not mature, and further research is needed to increase cell density. In addition, the optimization of the downstream process of virus production should also be strengthened, including physical and chemical parameters, Research on the mass production of human diploid cell (HDCs) and rabies vaccines should also be explored.

## 5 Using computational biology to simulate large-scale upstream process development

There will be inevitable differences in upstream production from laboratory simulation to industrial large-scale production, and the most intuitive change is the scale of the bioreactor (Figure 1A). In the manufacturing activities of bioreactors with a scale of more than 10,000 L, how to maintain the long-term stability of cell lines in bioreactors is currently the biggest problem facing upstream culture (Ueki et al., 2021). Obviously, it is not realistic to directly carry out large-scale production and optimization. The application of computational biology to upstream production can solve this dilemma to a great extent. The whole training process is simulated by a computer, various parameters are continuously tested and optimized, and the best model is established, which can theoretically realize the industrial upstream production with low cost and high efficiency.

Ueki et al. designed an inverted hybrid bioreactor with mutual mixing characteristics. They used Computational Fluid Dynamics (CFD) simulation software (FLUENT) to calculate the shear stress and vector of the reactor and rotating bioreactor, and used unsteady state and steady state analysis, respectively. At the same time, a κ-ε model was established for comparison. The results showed that the maximum shear stress and average shear stress of the inverted hybrid bioreactor were significantly lower than those of the conventional rotary hybrid bioreactor (Zhao et al., 2005). The application of the bioreactor can greatly reduce cell damage, thus affecting the cells’ physiological activity, and finally improve the maximum cell growth density (Nascu et al., 2021).

East China University of Science and Technology, Tongji University, and the University of Surrey proposed to use high-dimensional model representation (HDMR) to analyze and simulate the global sensitivity of perfusion bioreactor. The Contois parameter, the lactate Michaelis-Menten growth constan, the maximum consumption rate of glucose, the maximum concentration of glucose, the maximum consumption rate of oxygen, the maximum concentration of oxygen and cells death kinetic parameter were used as input parameters, and glucose concentration, oxygen concentration, lactic acid concentration, cell density, and cell growth were used as output parameters. They established a comprehensive mathematical model and carried out simulation using computational fluid dynamics software (COMSOL MultiPhysitics v5.5). The application of HDMR provides suitable physical and chemical parameters for cell growth (Bayrak et al., 2016). Another team focused on the relationship between the cell cycle and changes in the culture environment. Based on the agent-based modeling (ABM) method, they established a model that simulates the growth of individual mammalian cells and their circulatory regulation in response to changes in culture conditions (Ferreira et al., 2021). Such a model can predict the process of cell growth by inputting physical and chemical parameters, and its reliability can be verified experimentally.

In the face of the recent pandemic of COVID-19, a mathematical model has been established to simulate the production of adenovirus vaccine. Ferreira et al. designed, modeled, and economically evaluated the upstream production process of the new coronavirus vector vaccine using SuperPro Designer v12 software. The results showed that the virus titer produced by the perfusion culture method was 1 × 10^12^ VP/mL, and the final vaccine dose was 4 million doses per year (Trabelsi et al., 2019). In this experiment, computational biology was applied to vaccine production, and it proved theoretically that an adenovirus vector vaccine can be produced on a large scale and at low cost. However, the experiment did not integrate the early and late costs of vaccine development into the whole modeling system, which is also the main limitation of the model. However, to some extent, it provides valuable information for the development of adenovirus vaccines and speeds up the process of vaccine research and development.

In a word, the use of computer software to simulate the whole process of upstream culture and even vaccine production is expected to become the mainstream development direction in the future. Although the introduction of computational models can minimize resource consumption for vaccine research and development, the existing technology still has a large number of defects, resulting in many deviations between the theoretical value and the actual value. The biggest obstacle to the development of engineering-level bioreactor computing models is the lack of quantitative data and physical models. In the final analysis, human understanding of cells is still too shallow. At present, although computational biology has made many achievements in simulating virus production and cell culture, many computational models still remain at the level of reconstructing known experimental phenomena. Most models are forced to ignore the complex cell structure and simulate the cell state directly, which will cause marked deviations from the theoretical results when it comes to the interaction with external objects in reality. Therefore, computational biology requires further coordinated development with bioreactors to cope with the challenges brought by the differences in upstream production from the laboratory to the industrial scale.

## 6 Conclusion

It has been proven that vaccine supply is a key determinant of vaccination time, especially in low- and middle-income countries. To achieve highly process-intensive viral vaccine production, laboratory-scale research provides us with a lot of data and information to help with the choice of processes and reactors. For the further optimization of large-scale viral vaccine production, we must combine this large amount of data with new processes, such as the optimization of culture medium, the exploration of new culture schemes, the study of more efficient and safer cell lines, and the progress of automation technology. Currently, recently developed combinatorial technologies, such as metabonomics, genomics, and proteomics, might assist design techniques for high cell density growth and viral generation. The feeding or perfusion rate can also be directly controlled through on-line monitoring of substrate and metabolite concentrations. In addition, with the development of computational biology, studies have reported the use of software to establish bioreactor system models to simulate and economically evaluate vaccine production, which makes it possible to develop new vaccines more quickly in the face of new outbreaks of infectious diseases. Therefore, the rapid development and maturity of these technologies are of great significance to high efficiency and low-cost vaccine production, representing an important future research direction.
